# Current Challenges in Detecting Food Allergens by Shotgun and Targeted Proteomic Approaches: A Case Study on Traces of Peanut Allergens in Baked Cookies

**DOI:** 10.3390/nu4020132

**Published:** 2012-02-21

**Authors:** Romina Pedreschi, Jørgen Nørgaard, Alain Maquet

**Affiliations:** European Commission (EC), DG Joint Research Centre (JRC), Institute for Reference Materials and Measurements (IRMM), Geel 2440, Belgium; Email: romina.pedreschi@ec.europa.eu (R.P.); jorgen.norgaard@ec.europa.eu (J.N.)

**Keywords:** data dependent acquisition, peanut, food allergens, proteomics, shotgun, selective reaction monitoring, targeted

## Abstract

There is a need for selective and sensitive methods to detect the presence of food allergens at trace levels in highly processed food products. In this work, a combination of non-targeted and targeted proteomics approaches are used to illustrate the difficulties encountered in the detection of the major peanut allergens Ara h 1, Ara h 2 and Ara h 3 from a representative processed food matrix. Shotgun proteomics was employed for selection of the proteotypic peptides for targeted approaches via selective reaction monitoring. Peanut presence through detection of the proteotypic Ara h 3/4 peptides AHVQVVDSNGNR (*m/z* 432.5, 3+) and SPDIYNPQAGSLK (*m/z* 695.4, 2+) was confirmed and the developed method was able to detect peanut presence at trace levels (≥10 μg peanut g^−1^ matrix) in baked cookies.

## Abbreviations

ACN, acetonitrile; CE, collision energy; CHAPS, 3-[(3-cholamidopropyl)dimethylammonio]-1-propanesulfonate; DDA, data dependent acquisition; DIA, Data independent acquisition; DT, dwell time; DTT, dithiothreitol; MCP, microchannel plate; PBS, phosphate buffered saline; PTP, proteotypic peptide; Q-TOF MS, quadrupole time of flight mass spectrometer; SDS-PAGE, sodium dodecyl sulfate polyacrylamide gel electrophoresis; SRM, selective reaction monitoring; TBS, Tris buffered saline; UPLC, ultra performance liquid chromatography.

## 1. Introduction

An increasing incidence of food allergies in Europe and USA [1,2] is being reflected in clinical studies. European legislation recognizes so far 14 major allergenic foods [3] and requires a mandatory declaration when they are part of the ingredients; in cases where the manufacturer cannot exclude their presence as a result of accidental contamination in foodstuffs the label often contains the phrase “may contain”. But so far, there are no established threshold limits below which an allergen poses only a small risk of causing harm to an allergic consumer. Even though commonly accepted trigger levels have not been established yet, there is consensus among the scientific community, that allergen detection methods should be capable of covering the low ppm range (1–10 mg allergenic ingredient kg^−1^ food product).

Currently, to detect allergens in food products, enzyme-linked immunosorbent assays (ELISA) and PCR analyses were adopted as methods of choice by the food industry and official food control agencies [2,4]. However, food matrix and processing effects can result in large numbers of false positives and negatives using these methods [5], which lack the precision and rigor needed in cases of liability issues [6]. Mass spectrometry driven approaches on the other hand can be successfully used as a confirmatory platform given the specificity and sensitivity that can be achieved. In addition, multi-allergen detection and quantification is feasible after some stringent considerations are fulfilled [6].

The bottom up mass spectrometry approach (reconstruction of the protein based on peptides) either for characterization of the allergenic protein or by a targeted approach for sensitive detection/quantification are being used as platforms for allergen detection with promising outcomes [7,8].

The lack of established guidelines addressing different issues during sample preparation for analysis of food allergens makes it difficult to have a generic platform for multi-allergen detection and quantification. Published investigations in many cases lack experimental details and pose question marks relating to critical points of the food allergen detection workflows. It is of urgent necessity that certain aspects of the protocol used are stated: target analyte, source of allergenic food used (e.g., raw, roasted, defatted, *etc*.), incurred or spiked allergenic food (before or after processing); clear statement of reporting units (mass ratio of allergenic food, total proteins, allergenic protein target), digestion conditions (possible peptide modifications), *etc*., just to cite a few aspects of the sample preparation phase.

In this manuscript, we would like to illustrate with peanut serving as an example: (i) the challenges to be faced to detect food allergens at trace levels in complex food matrices (1–10 mg peanuts kg^−1^ cookie); (ii) the need for improvements in sample preparation and mass spectrometry analytical tools to achieve low levels of detection; and (iii) the awareness on key issues related to the development of a robust multi-allergen and quantitative method for trace analysis of food allergens.

## 2. Materials and Methods

### 2.1. Materials

Peanut included in the IRMM-481f peanut test material (mixture of five varieties, five different processing conditions) were utilized (Table 1). Wheat flour based cookie containing different amounts of IRMM peanut mixture (0, 10, 100, 1000 and 10,000 μg∙g^−1^ matrix) were prepared in house. Incurred cookies were baked at 180 °C for 16 min. The wheat flour based cookie recipe used consisted of: wheat flour 49.0%, butter 19.6%, dust sugar 18.4%, skimmed milk powder 5.9%, water 6.6%, sodium chloride 0.3%, sodium hydrogen carbonate 0.1%, ammonium bisulfate 0.1%. Baked cookies were grinded to a particle size <250 μm in liquid nitrogen and stored at −20 °C until use. Cookie dough was incurred with different amounts of peanuts before baking. Homogeneity of the cookie material was tested using ELISA kits.

**Table 1:** Detailed information of the IRMM-481 peanut test material used to prepare the incurred cookies.

Peanut variety, origin	Peanut treatment
Runners Argentina	Blanched air-roasted at 140 °C for 20 min
Common Natal, South-Africa	Raw, air-roasted at 160 °C for 13 min
Virginia, USA	Blanched, oil roasted at 145 °C for 25 min
Virginia, China	Blanched, oil roasted at 140 °C for 9 min
Jumbo Runners, USA	Blanched only

### 2.2. Chemicals

All chemicals used for sample preparation were purchased from VWR International (West Chester, PA, USA) and were at least analytical reagent grade. PlusOne chemicals for gel electrophoresis (Tris, glycine, 3-[(3-cholamidopropyl)dimethylammonio]-1-propanesulfonate (CHAPS), urea, thiourea, dithiothreitol (DTT), dimethylformamide), 2D clean up and 2D quantification kits were purchased from GE Healthcare (Uppsala, Sweden). Mini gels NUPAGE^®^ 12% Bis-Tris (1.0 mm) were obtained from Invitrogen (Carlsbad, CA, USA). Water from a Milli-Q water system (Millipore, Bedford, MA, USA) was used. Trypsin mass spectrometry grade (Cat. # 786-578) was obtained from G Biosciences (St. Louis, MO, USA).

### 2.3. Protein Extraction and Quantification

Tris buffered saline (TBS) extraction as previously described [5] was used. One to five g of cookie sample were extracted in 10 to 20 mL of TBS buffer (20 mM Tris, 150 mM NaCl, pH 7.4) at 4 °C in an ultrasonic bath for 20 min. After centrifugation at 3500 g for 30 min, proteins in the supernatant were quantified with the 2D Quant kit and further purified and precipitated with the 2D clean up kit following the instruction manuals of GE Healthcare (Uppsala, Sweden).

### 2.4. Protein Enrichment

A large and highly diverse bead-based library of combinatorial peptide ligands known as ProteoMiner™ (Biorad, MO, USA) was used for protein enrichment. The large sample loading capacity ProteoMiner™ protein enrichment kit was employed. One to five g of cookie samples incurred with peanuts (0, 10, 100, 1000 and 10,000 μg∙g^−1^ matrix) were extracted with TBS buffer. After equilibration of the columns as described in the kit, proteins were bound to the ligands for 3 h at room temperature under agitation. Samples were washed with phosphate buffered saline solution (PBS) composed of 150 mM NaCl, 10 mM NaH_2_PO_4_ at pH 7.4 following the instructions of the manufacturer. The unbound fraction was collected in a separate tube. The enriched sample was eluted with 8 M urea and 2% CHAPS according to the manufacturer’s instructions. For all cookie samples incurred with peanuts, SDS-PAGE was used to analyze the protein profiles of the initial extract, enriched and unbound fractions. Protein content was normalized to 5 μg per lane to allow comparisons among the different fractions. For mass spectrometry analysis, samples (initial extract, enriched and unbound fractions) were cleaned up and precipitated with the GE Healthcare kit described in the material and method section and further trypsin digested.

### 2.5. SDS-PAGE

Fifteen well mini gels NUPAGE^®^ 12% Bis-Tris (1.0 mm) from Invitrogen were loaded with 5 μg protein sample and run in a NUPAGE^®^ electrophoresis system (Invitrogen). Two gels were run at 200 V for approximately 50 min. Silver stained gels [9] were scanned and evaluated with Labscan software (GE Healthcare). Molecular weight standards of 3.5–260 kDa from Invitrogen and of 8–220 kDa from Sigma-Aldrich (USA) were run in parallel.

### 2.6. Gel Free and Gel Based Trypsin Digestion

Gel free trypsin digestion was performed as follows: Fifty micrograms of protein were dissolved in 50 μL of Rapigest™ (Waters, Manchester, UK) in a 50 mM ammonium bicarbonate solution. Two and a half microlitres of a 50 mM DTT solution in 50 mM ammonium bicarbonate was added and let to stand for 30 min at 60 °C under agitation. Samples were cooled down and 5 μL of a 100 mM iodoacetamide in 50 mM ammonium bicarbonate was added and let to stand for 30 min in darkness. One microlitre of a sequencing grade 1 μg∙μL^−1^ trypsin solution (1:50, enzyme:protein ratio) was added and let to incubate for 5 h at 37 °C. After incubation, 5 μL of 500 mM HCl solution was added and transferred to a molecular mass cut-off filtration device (3000 MWCO) and centrifuged at 14,000 g for 10 min. The filtrate was recovered for nano UPLC-Q-TOF-MS/MS analysis.

Gel bands were destained with 15 mM potassium ferrocyanide per 50 mM sodium thiosulfate solution and then reduced with 10 mM DTT in 50 mM ammonium bicarbonate for 30 min. Alkylation was done with 55 mM iodoacetamide in 50 mM ammonium bicarbonate for 30 min. Following, gel plugs were dehydrated with ACN in a vacuum concentrator until dryness and then trypsin (20 ng∙μL^−1^) was added and let to stand overnight at 37 °C. Peptides were extracted once with 1% formic acid and 2% ACN and twice with 50% ACN. After evaporation until dryness in a vacuum concentrator, peptides were re-suspended in 0.1% formic acid.

### 2.7. Nano LC Separation of Peptides

Separation of peptides was performed on a nano LC reversed phase chromatography system. A 180 μm × 20 mm Symmmetry C_18_ (5 μm) nano Acquity™ was used as trap column. Separation of peptides was carried out on a 75 μm × 100 mm (1.7 μm) BEH 130 C_18_ nano Acquity™ column. Eluent A was composed of 0.1% formic acid in water and eluent B was 0.1% formic acid in ACN. A flow rate of 0.4 μL∙min^−1^ was used. The linear gradient used to achieve separation of peptides was as follows: (initial) 97% A, 3% B; (0.5–80 min) 60% A, 40% B; (80–85 min) 15% A, 85% B; (85–90 min) 15% A, 85% B; (90–95 min) 97% A and 3% B and (95–100 min) 97% A and 3% B. One microlitre of a sample mixture of ~0.8 μg∙μL^−1^ prepared in Rapigest™ was injected for in liquid digested samples and 5 μL of the same sample mixture for gel digested samples.

### 2.8. Nano Electrospray Q-TOF Tandem Mass Spectrometry (Nano-ESI Q-TOF MS/MS)

MS/MS experiments were carried out in a quadrupole time of flight mass spectrometer (Q-TOF Ultima Global, Waters, Manchester, U.K) equipped with a nano-electrospray Z spray source. The operating conditions of the Q-TOF-MS were: Capillary voltage 3.0 kV, sample cone voltage, 100 V; source temperature, 80 °C. The instrument was operated in positive ion mode. Time of flight was operated in a continuous extraction mode. In the positive linear mode (V mode) an accelerating voltage of 9.10 kV was used for the TOF and the MCP value of the detector was set to 2200 V. A mass calibrant of Glu-Fibrinopeptide B (Sigma Aldrich) at 500 *f*mol∙μL^−1^ was utilized in MS/MS mode. Lock spray was utilized during the whole acquisition. Full scans (MS mode) were performed over the 350–1900 *m/z* range with scan time of 0.9 s and interscan time of 0.1 s. For peptide fragmentation, the instrument was used in a data dependent acquisition MS survey mode (MS/MS). Thus, the fragmentation of an ion is achieved when a minimum intensity (specified value) is detected (simultaneous fragmentation of the three most abundant ions). The collision energy was varied between 5 and 55 V according to the mass and charge state of the respective peptides.

### 2.9. Database Searching

The fragmentation ion spectra obtained from the MS survey mode were processed using Mass Lynx version 4.0 (Waters), a software that converts MS/MS raw data to peak lists. After centroiding and background subtraction, the generated PKL files obtained were used for Mascot (Matrix of science, London, UK) database searching against a customized peanut allergen and Swiss-Prot database. This customized allergen database consisted of Swiss-Prot sequences of: allergen Ara h 1 (P43237, clone P17 precursor), allergen Ara h 1 (P43238, clone P41B precursor), allergen Ara h 2 isoform (Fragment, Q7Y1C0), allergen Ara h 2.02 (Q8GV20), allergen II (Fragment, Q941R0), allergen Ara h 3/Ara h 4 (Q8LKN1), Ara h 3 Glycinin (Fragment, O82580), Ara h 4 Glycinin (Q9SQH7), allergen Ara h 5 Profilin (Q9SQI9), allergen Ara h 6 (Fragment, Q9SQG5), allergen Ara h 7 (Q9SQH1), allergen Ara h 8 (Q6VT83). A maximum of one missed cleavages were allowed. Peptide tolerance and MS/MS tolerance were set to 100 ppm and 0.1 Da respectively. Modifications on cysteine residues by carboxyamidation were set as fixed and a possible modification of methionine by oxidation was set as variable modification.

### 2.10. Selective Reaction Monitoring (SRM)

SRM data were acquired on a Waters Quattro Premier triple quadrupole coupled to a Waters nanoAcquity Ultra Performance system fitted with a Waters Symmetry 5 μm particle diameter C_18_ 180 μm × 20 mm trap column and a 75 μm × 150 mm (1.7 μm) BEH 130 C_18_ column. Peptides were eluted using a linear gradient of 1–35% B over 50 min at a flow rate of 0.3 μL∙min^−1^. Solvent A corresponded to 0.1% formic acid in milli-Q water and solvent B to 0.1% formic acid in ACN.

The mass spectrometer was operated in SRM mode. For ionization, 2.75 kV of capillary voltage, 35 V of cone voltage and 100 °C capillary temperature were used. The collision energy was calculated using the following formulas: CE = 0.044 * (*m/z*) + 5.5 for doubly charged ions and CE = 0.051 * (*m/z*) + 0.5 for triply charged ions. Selection of transitions (precursor/fragment combinations) was based on the DDA experimental data generated on an Ultima Global Q-TOF instrument (Waters). The SRM transitions monitored for Ara h 1, Ara h 2 and Ara h 3 are presented in Table 2. A Blast similarity search using the tool [10] was performed confirming the specificity of these peptides. The dwell time (DT) for each transition was 0.05 s.

**Table 2:** Selective Reaction Monitoring (SRM) transitions monitored for detection of Ara h 1, Ara h 2 and Ara h 3.

Peptides	Parention (*m/z*) (+)	Fragmention(s) (*m/z*)	CE ^a^
**Ara h 1**			
VLLEENAGGEQEER	786.9 (+2)	989.5 (y9)/875.4 (y8)/804.4 (y7)/747.3 (y6)/561.3 (y4)/304.2 (y2)	40
DLAFPGSGEQVEK	688.8 (+2)	1077.5 (y10)/930.5 (y9)/833.4 (y8)/447.2 (b4)/300.2 (b3)/229.1 (b2)	36
**Ara h 2**			
CCNELNEFENNQR	863.8 (+2)	1050.5 (y8)/807.4 (y6)/660.3 (y5)/531.3 (y4)	43
NLPQQCGLR	543.3 (+2)	858.4 (y7)/761.4 (y6)/633.3 (y5)/200.1 (a2)	29
CDLEVESGGR	561.2 (+2)	846.4 (y8)/604.3 (y6)/505.2 (y5)/376.2 (y4)	30
CMCELQQIMENQSDR	1006.9 (+2)	1721.8 (y14)/1361.7 (y11)/1248.6 (y10)/992.5 (y8)/879.4 (y7)/748.3 (y6)/ 619.3 (y5)/292.1 (b2)	49
**Ara h 3**			
LNAQRPDNR	361.9 (+3)	1083.6 (ymax)/970.5 (y8)/856.4 (y7)/657.4 (y5)/228.1 (b2)	19
SPDIYNPQAGSLK	695.4 (+2)	1389.7 (ymax)/1302.7 (y12)/977.5 (y9)/814.5 (y8)/700.4 (y7)/475.3 (y5)/300.1 (b3)	36
AHVQVVDSNGNR	432.5 (+3)	749.5 (b7)/663.3 (y6)/535.4 (b5)	23

^a^ CE: collision energy. Dwell time (DT) for each transition was 0.05 s.

## 3. Results and Discussion

Allergen detection methods must be sensitive and selective enough to detect the target proteins at levels as low as 1–10 mg allergenic food kg^−1^ food matrix. When investigating peanuts as the allergenic food source, given that they are largely incorporated in processed foods and thus a potential cross contact of intended peanut free products cannot be ruled out representing a constant risk to allergenic consumers [11]. In addition, detection of traces of peanut allergens in food products is difficult because they are obscured by the food matrix and processing hinders detection.

**Figure 1:**
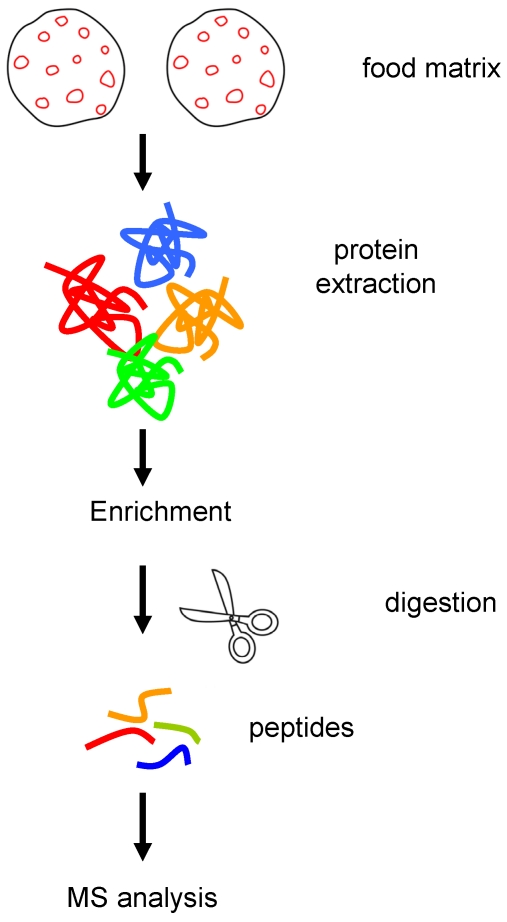
Workflow for sample preparation for mass spectrometry analysis. The different steps involve: protein extraction from the matrix, protein enrichment (optional), enzymatic (e.g., trypsin) digestion and MS analysis of the peptide mix either through shotgun (DDA) or targeted (SRM) proteomic approaches.

The workflow of Figure 1 is a typical approach followed to detect food allergens. It includes basic steps such as protein extraction, enrichment (if necessary), protein digestion and detection of peptides from the allergenic proteins through mass spectrometry platforms. To date there are eleven allergenic proteins in peanuts identified of which Ara h 1, Ara h 2 and Ara h 3 are recognized as the most abundant and major peanut allergens [12,13,14]. Ara h 1, Ara h 2 and Ara h 3 represent 12−16%, 5.9−9.3% and 21.8−38.5% of the total protein content determined by SDS-PAGE quantitative studies [12]. If the target is to detect 10 ppm of allergenic food kg^−1^ cookies the question arises, *how much is the quantity of the major allergens reaching the detector in the mass spectrometer?* *Since Ara h 3 is the most abundant peanut allergen across different varieties, all model calculations were done with this target.* It can be assumed that the average protein content of peanuts is 20%, and that Ara h 3 represents 30% of total peanut proteins. Five grams of cookies contaminated with 10 μg peanuts g^−1^ matrix are extracted with 20 mL of TBS buffer giving an average content of Ara h 3 of 2.45 *f*mol μL^−1^ solution. For protein digestion, 50 μg of total proteins (from the cookie matrix plus peanuts) are treated and approximately 0.8 μg total protein is loaded on the column which results in an ideal and theoretical content of 480 *a*mol Ara h 3. This is an optimistic scenario, since 100% recovery during protein extraction, complete digestion and 100% ionization efficiency of peptides is assumed. Therefore, *detection methods should be capable to detect targeted peptides in the *a*mol-low femtomol range*.

To complicate this scenario, the presence of other highly abundant proteins from the matrix (cookie) may hamper detection of the low abundant ones (trace amounts of target allergenic foods) by ion suppression of the target peptides. In this particular case, wheat flour and skimmed milk powder make up 50% and 6% of the cookie composition. Assuming total protein contents of 10% and 36% respectively for wheat flour and skimmed milk powder, we are talking about differences in protein or peptide levels of at least 6 orders of magnitude. *A reasonable question would be if current mass spectrometry technology is able to cope with such huge differences?* This will be discussed in the coming sections.

**Figure 2:**
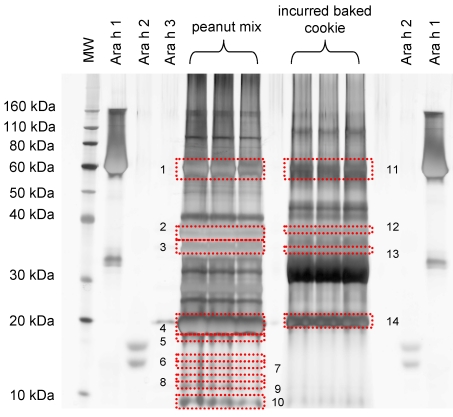
SDS-PAGE protein profiles for IRMM-481f peanut mix and 16 min baked cookie incurred with 100,000 μg IRMM-481f peanut mix g^−1^ matrix. The different band numbers were submitted to trypsin digestion and peptides submitted to nano LC-Q-TOF-MS/MS for protein identification. The list of peptides and identified proteins are presented in Table 3. Five micrograms protein was loaded per lane. A molecular weight standard (MW) of 3.5–260 kDa was run in parallel.

Up to now processing effects are not sufficiently being taken into account. Food processing may induce some unknown protein modifications in the allergen targets [15]. Figure 2 displays a SDS-PAGE protein profile for the IRMM-481f peanut mix used to prepare the incurred cookies and cookies incurred with high amounts of peanuts (100,000 μg∙g^−1^ matrix). The allergome of peanut is rather complex. Ara h 1 is a 63–68 kDa glycoprotein assembled in di and trimeric complexes. Of the allergen Ara h 2 two isoforms with masses of *ca.* 16 and 17 KDa have been isolated. Ara h 3 and Ara h 4 are isoallergens and can be designated as the allergen Ara h 3/4. The allergenic protein Ara h 3/4 consists of an acidic and basic subunit which remain covalently linked by an intermolecular disulfide bridge and associate into a very stable hexameric structure. The acidic subunit has a molecular mass in the range of 40–45 kDa, whereas the basic subunit has a mass of *ca.* 25 kDa. Ara h 3/4 is mainly proteolytically modified (truncation at multiple sites) with possible glycosylation. Proteolytic truncation was observed for the acidic subunit but not for the basic resulting in a series of polypeptides ranging from 13–45 kDa [16]. Ara h 1 and Ara h 3 were clearly identified through mass spectrometry in both samples (bands 1–4; 11 and 14; Table 3). Band 11 identified Ara h 1 but also the presence of alpha-casein which was detected and confirmed by mass spectrometry (Table 3). Co-migration of major allergens with matrix proteins was observed and thus interference with their detection at trace levels is expected. Band 13 was negative for Ara h 3 and identified casein instead, while Ara h 2 (bands 5 and 7) could not be detected in the processed food product even when the concentration of added peanut was very high. Ara h 1 and Ara h 3 are present in higher concentrations than Ara h 2 in the different peanut varieties [12]. IRMM-481f peanut mix presents a low amount of Ara h 2 which limited its qualitative detection by SDS-PAGE and could not be detected even when the matrix contained very high concentrations of peanuts. *Is it because of the low abundance of this protein and low sensitivity of the gel based approach or are there any interactions with other matrix components that resulted in protein modification and thus protein migrate differently and consequently was not identified by MS analysis?* These are certainly issues that need to be answered.

**Table 3:** Tryptic peptides identified from IRMM peanut mix and 16 min baked cookies incurred with 100,000 μg peanuts g^−1^ matrix of IRMM peanut mix.

Protein identification	Band # ^a^	Peptides matched	Mascot Score
**IRMM peanut mix**			
Ara h 1 clone P41B precursor	1	18, 15, 26	756, 744, 1473
Ara h 1 clone P17 precursor		16, 15, 29	704, 726, 1619
Allergen Ara h 3 Ara h 4		1, 3, 9	81, 76, 509
Allergen Ara h 3 Ara h 4	2	13, 7, 12	637, 369, 728
Ara h 3 Glycinin	3	1, -, 1	90, -, 70
Allergen Ara h 3 Ara h 4	4	6, 11, 9	365, 771, 576
Allergen Ara h 2	5	3, 3	140, 114
Ara h 3 Glycinin	6	2	98
Allergen Ara h 3 Ara h 4	7	2	96
Ara h 1 clone P17 precursor		2	67
Ara h 1 clone P41B precursor		2	67
Allergen Ara h 2 isoform		1	51
Allergen Ara h 6	8	6	209
Allergen Ara h 3 Ara h 4	9	8	431
Allergen Ara h 6		10	369
Allergen Ara h 3 Ara h 4	10	10	558
**Cookie incurred at 100,000 μg∙g^-1^**			
Ara h 1 clone P41B precursor			
Ara h 1 clone P17 precursor	11	14, 10, 8	766, 582, 481
Allergen Ara h 3 Ara h 4		13, 11, 10	686, 569, 552
		4, 4, 2	264, 190, 134
Allergen Ara h 3 Ara h 4			
	12	3, 1, 1	103, 20, 50
Alpha casein S2			
	13	3, 3	102, 117
Allergen Ara h 3 Ara h 4			
Alpha casein S2	14	6, 7	340, 524
		(−), 2	(−), 103

^a^ Band numbers correspond to those reported in Figure 2. When sufficient amount of sample, three independent replicates were submitted to DDA MS/MS protein identification (three corresponding values for peptides matched, Mascot score and expect value) otherwise one or two samples were used. (−) Stands for a negative hit. The expect value is the probability to obtain a random protein identification. Expect values were in all cases <0.0001 peanuts g^−1^ matrix.

### 3.1. Shotgun Proteomics Approach: Nano LC-Q-TOF-MS/MS

Protein extracts from incurred cookies with 0, 10, 100, 1000 and 100,000 μg peanuts g^−1^ matrix were digested with trypsin and the peptide mixture was submitted to data dependent acquisition (DDA) with a Q-TOF instrument. Results are presented in Table 4. Ara h 1 was detected in samples containing at least 10,000 μg peanuts g^−1^ matrix. Ara h 3 was detected in samples containing ≥1000 μg∙g^−1^.

**Table 4:** DDA MS/MS results for 16 min baked cookies incurred with different amounts of peanuts (0–100,000 μg g^−1^ matrix).

Amount of peanut μg∙g^−1^ matrix	Protein Identification	Peptides	*m/z*	charge
**1**	Negative			
**10**	Negative			
**100**	Negative			
**1000**	Ara h 3/Ara h 4	LNAQRPDNR	362	3+
	Ara h 4 Glycinin	AHVQVVDSNGNR	433	3+
	Ara h 3 Glycinin			
**10,000**	Ara h 3/Ara h 4	LNAQRPDNR	362	3+
	Ara h 4 Glycinin	AHVQVVDSNGNR	433	3+
	Ara h 3 Glycinin	SPDIYNPQAGSLK	695	2+
	Ara h 1 clone P17	VLLEENAGGEQEER	787	2+
	Ara h 1 clone P41B	DLAFPGSGEQVEK	689	2+
		DQSSYLQGFSR	644	2+
		GTGNLELVAVR	565	2+
**100,000**	Ara h 3/Ara h 4	LNAQRPDNR	362	3+
	Ara h 4 Glycinin	AHVQVVDSNGDNR	433	3+
	Ara h 3 Glycinin	SPDIYNPQAGSLK	695	2+
		FNLAGNHEQEFLR	788	2+
		GENESDEQGAIVTVR	802	2+
		FFVPPSEQSLR	654	2+
		TANDLNLLILR	628	2+
		RPFYSNAPQEIFIQQGR	684	3+
	Ara h 1 clone P17	VLLEENAGGEQEER	787	2+
	Ara h 1 clone P41B	DLAFPGSGEQVEK	689	2+
		DQSSYLQGFSR	644	2+
		GTGNLELVAVR	565	2+
		WGPAEPR	407	2+
		QFQNLQNHR	593	2+
		SSDNEGVIVK	524	2+
		GSEEEDITNPINLR	794	2+
		DGEPDLSNNFGR	661	2+
		IFLAGDKDNVIDQIEK	607	3+
		EGEQEWGTPGSHVR	524	2+
		SSENNEGVIVK	588	2+
		LFEVKPDK	488	2+
		EGALMLPHFNSK	672	2+

Peptides were selected based on E-values lower than 0.05. *E*-value is the probability of a random peptide identification. Results are based on three independent replicates.

Ara h 2 could not be detected even in samples containing 100,000 μg peanuts g^−1^ matrix. The differences in detectability of the three major allergens in the matrix seem to be related to their initial content in the peanut source. Ara h 3 is the most abundant protein followed by Ara h 1 and Ara h 2 [12]. Baked cookies incurred with trace amount of peanuts are an excellent representation of a processed complex food matrix where huge differences in the content of individual proteins (food allergens *vs.* bulk proteins from other ingredients) can be expected. In addition, not all proteins generate a sufficient amount of detectable peptides: poor ionization and/or fragmentation behaviour are main issues that prevent peptide detection with the state-of-the-art mass spectrometry technology, which is even more accentuated when very complex matrices are used [15]. A way to circumvent the huge differences in concentration of proteins/peptides in the sample and ion suppression is to enrich the targeted analytes [15,17,18]. There is resilience for the use of enrichment techniques in trace analysis not only because of the expense of time and work but because they might complicate quantitative analysis. However it was the strategy taken in this work, otherwise it was not feasible to attain the required detection limits.

**Figure 3:**
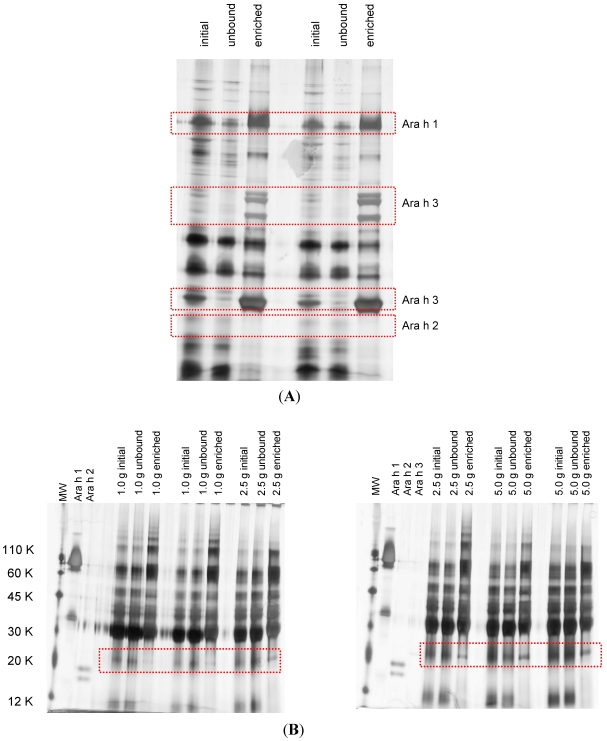
Enrichment of major peanut allergens through peptide ligand libraries (ProteoMiner™). (**A**) IRMM-481f peanut mix sample; (**B**) 16 min baked cookies incurred with 1000 μg IRMM-481f peanut mix g^−1^ matrix. Different amounts of starting material were extracted. Protein loaded was normalized to 5 μg per lane to allow comparisons. Initial corresponds to whole protein extract.

In the present study, the shotgun approach (data dependent acquisition—DDA) was limited by the required rate to carry out a precursor scan and fragmentation event for a single peptide, which was not fast enough to analyze all peptides. Consequently, bias towards highly abundant peptides was observed. Thus, a simple protein enrichment protocol based on a peptide ligand library known as ProteoMiner™ was evaluated. First, a possible enrichment of the major peanut allergens was confirmed in the peanut material used to prepare the incurred cookies (IRMM-481f peanut mix). Ara h 1 and Ara h 3 were satisfactorily enriched in the IRMM peanut mix sample (Figure 3A). Ara h 2, however, was not satisfactorily enriched and seemed to be washed away (present in the unbound fraction). A different situation was observed when the matrix was incorporated (baked cookie), only enrichment of Ara h 3 could be evidenced through SDS-PAGE and was dependent on the amount of the extracted material (Figure 3B). At least a 10× enrichment for Ara h 3 from cookies was achieved. In cookies incurred with 100 μg peanuts g^−1^ matrix, Ara h 3 could be detected after enrichment through LC-Q-TOF MS/MS (Table 5). Previous works in allergen detection have made used of enrichment techniques such as ultrafiltration, precipitation, and peptide ligand libraries [12,19,20]. Enrichment can increase the identification rate of the low abundant fraction proteins/peptides but the intrinsic limitation of a shotgun approach will remain [21].

**Table 5:** DDA MS/MS results for 16 min baked cookies incurred with trace amounts of peanuts (0–1000 μg∙g^−1^ matrix) after protein enrichment via peptide ligand library—ProteoMiner™.

Amount of peanut μg∙g^−1^ matrix	Protein identification	Peptides (*m/z*, charge)	MS/MS
**10**	Negative		
**100**	Ara h 3/Ara h 4	LNAQRPDNR (361.9, 3+)	1, 1, 1
	Ara h 4 Glycinin		
	Ara h 3 Glycinin		
**1000**	Ara h 3/Ara h 4	LNAQRPDNR (361.9, 3+)	2, 1, 1
	Ara h 4 Glycinin	AHVQVVDSNGDNR (432.5, 3+)	1, 1, 1
	Ara h 3 Glycinin	SPDIYNPQAGSLK (695.4, 2+)	1, 1, 1
		QIVQNLR (435.8, 2+)	1, 1, 1
		GENESDEQGAIVTVR (802.4, 2+)	1, 1, 0

Peptides were selected based on *E*-values lower than 0.05. *E*-value is the probability of a random peptide identification. Results are based on three independent extractions.

It is worth noting the importance of a shotgun approach. Indeed a targeted approach such as selective reaction monitoring (SRM) is developed based on experimental data obtained through a shotgun approach (e.g., data dependent acquisition) for selection of the proteotypic peptides. In addition, a shotgun approach is useful to assess unexpected or known protein/peptide modifications which might be frequently encountered when working with processed foods.

### 3.2. Targeted Proteomics Approach: SRM

SRM is a highly sensitive approach to selectively detect and quantify peptides previously selected and relying on the monitoring of specific ion transitions [22].

**Figure 4:**
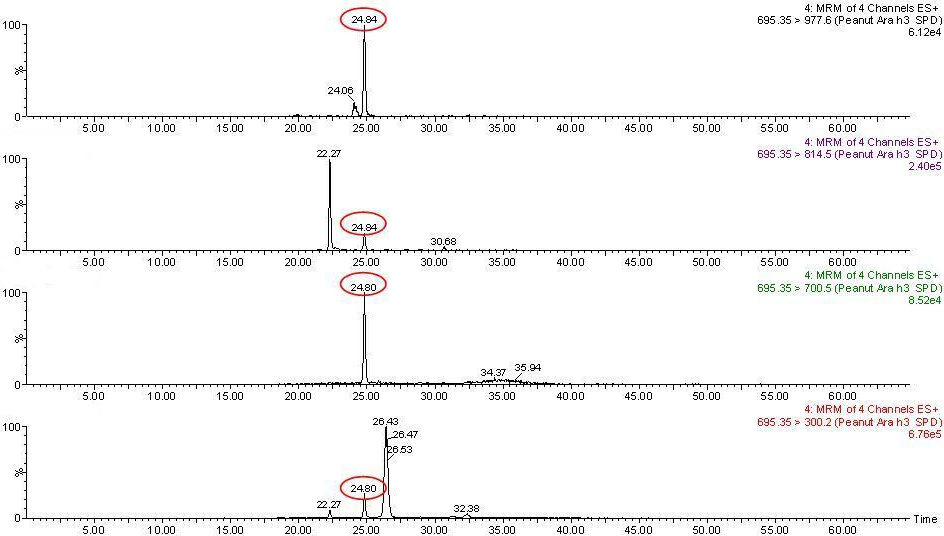
Selective Reaction Monitoring (SRM) of the Ara h 3 peptide SPDIYNPQAGSLK (*m/z* 695.4, 2+) in enriched cookie samples incurred with 10 μg peanuts g^−1^ matrix. SRM analysis was carried out on a Waters Quatro Premier triple quadrupole.

Selection of SRM transitions to be monitored was based on experimentally obtained data with a DDA approach in an ESI-Q-TOF instrument (Table 2). Peptides highly observable and detectable for Ara h 1, Ara h 2 and Ara h 3 were selected. Fragment ions were selected based on MS/MS spectra of these highly observable precursor ions (Table 2). At least three transitions per peptide were monitored to facilitate reliable identification of the peptide. Previously enriched incurred cookie samples (1, 10 and 100 μg peanuts g^−1^ matrix) were submitted to SRM. Two proteotypic peptides from Ara h 3 were detected in enriched cookie samples containing ≥10 μg peanuts g^−1^ matrix. These peptides corresponded to: AHVQVVDSNGNR (*m/z* 432.5, triply charge ion) from which three out of the three fragment ions were detected and SPDIYNPQAGSLK (*m/z* 695.4, doubly charged ion, Figure 4) from which 4 out of 7 fragment ions were detected. However, Ara h 1 was only detected in samples containing 100 μg peanuts g^−1^ matrix (DLAFPGSGEQVEK, *m/z* 688.8 doubly charged ion) and Ara h 2 peptides were not detected in the enriched samples tested. In the present study, a gain of a factor of ten in the average signal was achieved by fractionation/enrichment techniques previous to SRM analysis as already reported by Picotti *et al*. analysing yeast samples [23].

Previous SRM investigations focused on detection of peanut allergens in different food matrices have been reported [7,19,20,24]. However, one should be critical with issues concerning sample preparation and analysis that pose some reasonable questions on achieved detection limits. In the study of Careri *et al*. [19], for instance, the different food matrices tested (rice crispy and cacao based snacks) were fortified with different amounts of raw peanuts and it was not clearly reported if the different amount of peanut proteins were added before or after processing. Thus, they were dealing with spiked samples and raw peanuts. The effect of food processing on the extractability of Ara h 2 and Ara h 3/4 was not considered. The detection limits of 5 μg protein g^−1^ matrix for Ara h 2 and 1 μg protein g^−1^ matrix for Ara h 3/4 might be too optimistic. In addition, limits of detection were expressed as μg protein g^−1^ matrix which is different than μg peanuts g^−1^ matrix. In our study, we achieved detection of Ara h 3/4 in incurred cookies with 10 μg peanuts g^−1^ matrix targeting the same Ara h 3 peptides (*i.e.*, AHVQVVDSNGNR and SPDIYNPQAGSLK) reported by Careri *et al*. [19]. For Ara h 2, the same peptides reported by Careri *et al*. [19] CCNELNEFENNQR (*m/z* 863.8, doubly charged ion) and CMCELQQIMENQSDR (*m/z* 1006.9, doubly charged ion) were also targeted but unsuccessfully in our study even though an alkylation step was introduced. In our case, to confirm detection of a certain peptide in SRM mode at least three fragment ions needed to be detected as to be considered a true hit while in the case of Careri *et al*. [19] it was based on one fragment ion which could render confirmation of certain peptide doubtful. At least two fragment ions should be used: one as quantifier and the other as qualifier fragment ions. The ratio quantifier/qualifier must remain constant throughout samples and it is considered as another quality control step in SRM [7,8]. In the study of Shefcheck *et al*. [20], detection levels of 2 μg protein g^−1^ matrix (Ara h 1) from dark chocolate with SRM have been reported. The Ara h 1 peptides VLLEENAGGEQEER (*m/z* 786.9, doubly charged ion) and DLAFPGSGEQVEK (*m/z* 688.9, doubly charged ion) were targeted detecting three fragment ions per peptide. With this particular matrix, the most important factor affecting the extraction of the target analyte(s) seemed to be the interaction protein-tannins. The range in terms of concentration between the target analyte(s) and matrix proteins do not seem to be the major problem for detection since the sample was dark chocolate (proteins present belong to the cacao matrix). In cookie as food matrix, not only the huge range in terms of protein concentrations but the interaction and processing effects seem to be the limiting factors for detecting at least Ara h 1 and Ara h 2 at lower levels.

In a recent study conducted by Heick *et al*. [7,24] targeting seven different allergens from milk, egg, soy, hazelnut, peanut, walnut and almond in flour and bread as food matrices, detection limits of 11 μg∙g^−1^ matrix have been reported for the allergens from peanuts (Ara h 1 and Ara h 3/4, respectively). However, this detection limit is based on spiking different amounts of semi purified peanut proteins in blank matrix. Unfortunately, the source of peanut material was not described (e.g., raw or processed peanut, one variety or a mix or varieties representative of what is commercially available and used by the food industry) which might explain the differences in the selected proteotypic peptides. With the exception of DLAFPGSGEQVEK (*m/z* 688.8, doubly charged ion) from Ara h 1, the other three peptides largely differed from the ones selected in the present study. For example, the proteotypic peptide RPFYSNAPQEIFIQQGR (*m/z* 684.5, triply charged ion) from Ara h 3/4 was reported as the most intense marker [7]. However, in our study, we could only detect this peptide when the amount of peanuts was very high (>10,000 μg g^−1^ matrix) in the cookie matrix. The reported LOD value of 11 μg∙g^−1^ matrix might also be very optimistic since the starting material was defatted peanut, and peanut contains approximately 50% fat and the calculated LOD was based on matrix spiked with total peanut proteins.

There is consensus in the community that SRM is the approach to take for trace level detection of major food allergens, especially as confirmatory method when liability issues are raised. However, still aspects remain that need to be common practice and improved before a solid detection method is developed. Recommendations of the scientific community [6] working on the subject includes but are not limited to: history and description of the allergen food source, nature of the reporting units, quality control of MS data, *etc*.

### 3.3. Perspectives

Previous evaluations of the reproducibilty of ProteoMiner enrichment opens perspectives for its use in food allergen quantitation [25,26]. The quantitative reproducibility of cytokines per trial and not only in terms of enrichment factor was confirmed by immunodetection and nano LC-MS/MS [25]. A method that incorporated ProteoMiner enrichment has been successfully used for label free quantification of series of cerebrospiral fluid samples processed in parallel [26]. The signal intensity of peptides coming from growing amounts of exogenous spiked proteins in the matrix was evaluated. 

Data independent acquisition (DIA) known as MS^E^ presents itself as a promising approach to detect trace levels of food allergens in complex food matrices due to the higher sensitivity that can be achieved compared to the classical DDA approach. An advantage of MS^E^ is the possibility to account for certain modifications simultaneously, higher sequence coverage, and label free quantification [27]. Wei *et al*. [28] performed data independent acquisition (DIA) or MS^E^ to analyze Ara h 1 detectable peptides in raw and roasted peanuts. The potential of certain Ara h 1 peptides as markers was based on their presence in both raw and roasted peanuts at relatively high intensities. We detected the same Ara h 1 peptides: DLAFPGSGEQVEK and VLLEENAGGEQEER as the most abundant ones through DDA (Table 3). Wei and co-workers also introduced a matrix (*E. coli* tryptic digest) in a 1:200 v/v (Ara h 1 matrix) proportion and were able to perform label free Ara h 1 quantification with the introduction of an ADH tryptic digest as internal standard. However, this material does not represent a processed food product. In the present work, a much higher dynamic range was encountered in terms of concentration of proteins besides that a representative matrix (cookie) was incorporated. This MS^E^ approach has been explored by our team, but so far no comparable or better sensitivity has been achieved than the ones achieved with SRM.

Multi allergen detection and quantification are hot topics nowadays. An LC-MS based method claiming detection of seven different food allergens at trace levels in bread has been recently reported [7]. The LOD values reported need to be considered with caution since the allergen food sources are not fully described and might not be representative of real scenarios, being by far too optimistic. However, it is a first attempt that brings new insights into this possible multi allergen detection. For absolute allergen quantification, the topic is much more sensitive and we should be cautious on how to interpret quantification. Ideally, an isotopically labeled target protein that can be incorporated during the sample preparation workflow or even before processing simulating real processing and extraction conditions should be used. However, so far it is more realistic to use isotopically labeled peptides [6]. The digestion step for quantification of proteins/peptides is a key aspect and requires being reproducible and complete [29]. Assessment of the stability of target peptides (e.g., possible deaminations) during the proteolysis and afterwards is also of key importance for absolute protein quantification. Absolute quantification of milk allergens in a variety of food matrices has been recently reported [8]. Many of the target peptides present Q and N in their sequence. We have experimentally observed with our target peptides that when these amino acids are present they can undergo deamination to a certain degrees. Of course, this issue might be circumvented by accounting for the amidated and de-aminated forms in the SRM method. Given the different physico-chemical properties of the different allergenic proteins to target, the complexity of the different potential food matrices encountered and the diversity of food processing conditions, a generic sample preparation platform might not be realistic. However, it might be feasible to rely on a few number of sample preparation workflows grouping food products sharing similar characteristics (e.g., high protein content, high polyphenol content, *etc*.). This would certainly help for validation and standardization of food allergen detection methods through SRM targeted approaches.

## 4. Concluding Remarks

A well described and representative food allergen source (IRMM-481f), which is a mix of five commercially available different peanut varieties and five different processing conditions, was used contrary to previous reported investigations in which the food allergen source is vague or not representative enough. A representative and highly processed food matrix (cookies) with a high protein content of non target proteins (milk and wheat) was employed. This matrix is representative of what is to be expected when analyzing food samples contaminated with allergens: a wide range in protein concentration and a highly processed sample. This work mainly focused on method development through shotgun proteomics and sample preparation for future targeted confirmatory approaches such as SRM. The SRM approach followed, allowed us to detect Ara h 3 peptides at levels as low as 10 μg peanuts g^−1^ cookies. The two proteotypic Ara h 3/4 peptides AHVQVVDSNGNR and SPDIYNPQAGSLK were confirmed as markers of peanut presence in cookies containing as low as 10 μg peanuts g^−1^ cookies. This targeted SRM is being further optimized by our group to allow detection, confirmation and potential quantification of Ara h 3 and Ara h 1 peanut allergens in different food matrices.
